# Shotgun Metagenomics of Deep Forest Soil Layers Show Evidence of Altered Microbial Genetic Potential for Biogeochemical Cycling

**DOI:** 10.3389/fmicb.2022.828977

**Published:** 2022-03-01

**Authors:** Beat Frey, Gilda Varliero, Weihong Qi, Beat Stierli, Lorenz Walthert, Ivano Brunner

**Affiliations:** ^1^Forest Soils and Biogeochemistry, Swiss Federal Institute for Forest, Snow and Landscape Research (WSL), Birmensdorf, Switzerland; ^2^Centre for Microbial Ecology and Genomics, Genetics and Microbiology, University of Pretoria, Pretoria, South Africa; ^3^Functional Genomics Center Zurich (FGCZ), ETH Zürich/University of Zurich, Zurich, Switzerland

**Keywords:** forest, C and N cycles, CAZy, subsoil, metagenomics

## Abstract

Soil microorganisms such as Bacteria and Archaea play important roles in the biogeochemical cycling of soil nutrients, because they act as decomposers or are mutualistic or antagonistic symbionts, thereby influencing plant growth and health. In the present study, we investigated the vertical distribution of soil metagenomes to a depth of 1.5 m in Swiss forests of European beech and oak species on calcareous bedrock. We explored the functional genetic potential of soil microorganisms with the aim to disentangle the effects of tree genus and soil depth on the genetic repertoire, and to gain insight into the microbial C and N cycling. The relative abundance of reads assigned to taxa at the domain level indicated a 5–10 times greater abundance of Archaea in the deep soil, while Bacteria showed no change with soil depth. In the deep soil there was an overrepresentation of genes for carbohydrate-active enzymes, which are involved in the catalyzation of the transfer of oligosaccharides, as well as in the binding of carbohydrates such as chitin or cellulose. In addition, N-cycling genes (NCyc) involved in the degradation and synthesis of N compounds, in nitrification and denitrification, and in nitrate reduction were overrepresented in the deep soil. Consequently, our results indicate that N-transformation in the deep soil is affected by soil depth and that N is used not only for assimilation but also for energy conservation, thus indicating conditions of low oxygen in the deep soil. Using shotgun metagenomics, our study provides initial findings on soil microorganisms and their functional genetic potential, and how this may change depending on soil properties, which shift with increasing soil depth. Thus, our data provide novel, deeper insight into the “dark matter” of the soil.

## Introduction

In agricultural and forested areas, studies on soil microbial communities rarely include soil depths exceeding 30 cm (Yost and Hartemink, 2020). Most of the organic C in soils from various biomes is located in the topsoil at 0–30 cm soil depth (Crowther et al., 2019). Jobbágy and Jackson (2000) estimated in a worldwide study that 50% of the organic C in forests is in the top 20 cm. Xu et al. (2013) similarly calculated that about 70% of the soil microbial C and N in forested ecosystems is located in the upper 30 cm of the soils. In a few recent studies on soils, however, measurement went beyond the topsoil and included the deep soil down to a depth of 1 m in investigations of extracellular enzyme activities (Dove et al., 2020) or metagenomic attributes of novel bacterial taxa (Brewer et al., 2019). However, studies of the soil microbiome including soils at depths greater than 1 m are still particularly rare, leaving this soil compartment largely unexplored as a *terra incognita* (Andújar et al., 2017).

Deep soil layers harbor poorly known bacterial and fungal communities (Brewer et al., 2019; Robin et al., 2019; Liu et al., 2020; Frey et al., 2021). It is assumed that living conditions in deeper soils for soil organisms and plant roots are harsher in deeper soils, as a result of higher soil density, lower oxygen concentrations, and lower carbon (C) and nutrient availability (Lennon, 2020). Because soil processes, soil properties, and microbial communities are depth-dependent, soils should be studied at greater depths for a more comprehensive understanding of their relationship and interaction (Yost and Hartemink, 2020). In addition, it is important to understand deep soil microbial communities because deep soils are poorly accounted for in current models of biogeochemical processes.

Microorganisms are the key drivers of both C- and nutrient-cycling processes in the soil (Falkowski et al., 2008). Most soil microorganisms in forest ecosystems gain their energy by decomposing different types of C from soil organic C (SOC), which vary in degradability and require different mechanisms for decomposition (Lladó et al., 2019). Carbon and nutrients typically are more available in the topsoil, mainly because of larger input of leaf and fine root litter and root exudates, as well as higher biotic activity (Eldridge et al., 2016; Liu et al., 2020). Consequently, microbial biomass per unit soil mass is usually one to two orders of magnitude lower in the deep soil than in the topsoil (Eilers et al., 2012; Spohn et al., 2016). Nevertheless, the total biomass of Bacteria and fungi inhabiting deeper soil horizons can be as large as in the topsoil, and these microorganisms play similarly important roles in biogeochemical processes (Brewer et al., 2019), although we know very little about the microbial genetic potential including soil C and nutrient cycling, soil formation, iron redox reactions, and pollutant degradation.

Because C usually occurs in lower amounts in deeper soil layers compared to the topsoil, chemolithotrophic processes could become prominent in deep soil layers. This means that microorganisms could gain their energy from the oxidation of inorganic compounds instead of organic compounds (Kelly and Wood, 2013). The most common substrates that are reduced are nitrogen (N), sulfur and iron compounds as well as hydrogen (Thiel, 2011). Besides of inorganic C, oxygen concentrations also tend to be lower in deeper soil layers. Thus, anaerobic processes can also occur, where energy is generated through the reduction of nitrate, nitrite or sulfate (Hooper and Dispirito, 2013). Some microorganisms are also able to use CO_2_ as a C source. In this case, the resulting products are methane and acetic acid (Hooper and Dispirito, 2013). Ammonia-oxidizing Bacteria (AOB) and ammonia-oxidizing Archaea (AOA) are central players in the global N cycle. These microbial groups can perform the first part of the nitrification process, ammonia oxidation. This is an important rate-limiting step in the various N-cycling processes, including N fixation, mineralization, nitrification, and denitrification (Kowalchuk and Stephen, 2001; Zhang et al., 2013). Since AOB gain only little energy by oxidizing ammonia, a lower soil NH_4_^+^-N content in the deep soil leads to a decrease in AOB abundance, whereas AOA appear to be less constrained by the availability of N substrates and prosper in oligotrophic environments in the deep soil (Martens-Habbena et al., 2009; Trivedi et al., 2019).

In deeper soil layers, weathering processes can additionally contribute to the presence of essential nutrient elements such as Mg, P and Fe, through the mineralization of the bedrock (Uroz et al., 2009, 2015; Xi et al., 2018). Bacteria and fungi play a role in rock weathering by producing organic acids, hydrogen cyanide or siderophores (Frey et al., 2010; Brunner et al., 2011; Wongfun et al., 2014; Xi et al., 2018; Wang et al., 2019). In addition, specific environmental enzymes involved in S and Fe metabolisms could be relevant (Jiang et al., 2019; Li et al., 2020). Genes related to such weathering processes could be those involved in oxalate biosynthesis, in cyanide synthesis, and in siderophore synthesis and transport (Varliero et al., 2021).

In a first survey of the deep soil horizons in Swiss beech and oak forests, we observed the occurrence of poorly known bacterial taxa belonging to Nitrospirae, Chloroflexi, Rokubacteria, Gemmatimonadetes, and Firmicutes (Frey et al., 2021). Furthermore, archaeal phyla such as Thaumarchaeota and Euryarchaeota were more abundant in the deep soil than in the topsoil. Previous research has indicated that members of Chloroflexi might be adapted to nutrient-poor environmental conditions (Rime et al., 2016; Adamczyk et al., 2019) whereas Gemmatimonadetes might be specifically adapted to low-moisture availability (DeBruyn et al., 2011; Rime et al., 2015). It is noteworthy, that some members of Euryarchaeota produce methane (Bodelier and Steenbergh, 2014), but also oxidize methane, fix N, and reduce nitrates (Cabello et al., 2004).

The aim of the present study was to improve our understanding of the metabolic capabilities of the microbial communities in deep soil layers. While we focused on changes in taxonomic composition with increasing soil depth in our first survey (Frey et al., 2021), here we used shotgun metagenomics to assess changes in microbial C- and N-cycling potential and link these data to the properties in deep soil layers. In particular, we hypothesized that in deep soil layers: (1) the genus of the dominant tree species in a site does not play a crucial role in shaping microbial metabolic capabilities in soil, (2) the biogeochemical and chemolithotrophic capabilities of the microbial communities are enhanced relative to shallower soil, and (3) soil N cycling is particularly affected by the metabolic capabilities of the microbial communities. We specifically focused on genes in the Carbohydrate-Active enZymes (CAZy) database, which are responsible for the catabolism of various C-complexes with varying decomposability, ranging from labile C (e.g., monosaccharides and polysaccharides) to recalcitrant C (e.g., lignin). Our study is one of the first to characterize the functional genetic potential and metabolic capabilities of microorganisms in deep soil layers and contributes to an integrated perspective on the soil microbial communities of beech and oak forests.

## Materials and Methods

### Forest Sites

The six investigated forest sites are located in the relatively warm and dry Rhone Valley (Chamoson and Saillon) and in the Jura Mountains (Neunkirch) of Switzerland with mean annual precipitation sums of 815–955 mm and mean annual temperatures of 8.5–9.4°C (Supplementary Table 1; Remund et al., 2014). The sites are covered mainly by European beech (*Fagus sylvatica* L.) or by oak species (*Quercus* spp.), with oak sites having warmer and drier soils than European beech sites (see also Frey et al., 2021). All of the forest stands have a near natural tree species composition originating from natural regeneration and have been unmanaged for several decades (Frey et al., 2021). The forest sites are part of the research platforms WSL Forest Soil Database. The soil classification (Supplementary Table 1) used in this study followed the system of the Food and Agriculture Organization of the United Nations (IUSS Working Group WRB, 2007).

### Soil Sampling and Soil Chemical and Physical Analyses

A soil profile down to 2 m depth was excavated in the center of a group of dominant trees at each study site in spring 2014. Three soil samples per soil depth were taken with 1 L steel cylinders (10.7 cm height and 11.1 cm diameter) at depths of 0–10 cm (L1), 15–25 cm (L2), 45–55 cm (L3), 75–85 cm (L4), 110–125 cm (L5), 140–155 cm (L6), and 180–200 cm (L7) (for details see Frey et al., 2021). For soil chemical and physical analyses, approximately 3–4 kg of soil was sampled separately from the various depths (L1–L7). All soil samples were then transported in plastic bags to the laboratories and stored at 4°C until further processing. The soil samples for chemical and particle size analyses were dried a 60°C and sieved through a 2 mm mesh sieve. Soil pH was determined in 0.01 M CaCl_2_. For C and N analyses, an aliquot of the dried and sieved samples was ground for 3 min using a vibrating ball mill (MM2000, Retsch, Haan, Germany) with zircon-grinding tools. Organic C (*C*_org_) and total N (*N*_tot_) were determined with a CN elemental analyzer (NC2500; CE Instruments Ltd., Hindley Green, United Kingdom) whereas inorganic carbonates were removed by HCl fumigation of the samples prior to combustion (Walthert et al., 2010). Exchangeable cations (Na, K, Mg, Ca, Mn, Al, and Fe) were extracted (in triplicate) in an unbuffered solution of 1 M NH_4_Cl. The effective cation-exchange capacity (CEC) was obtained by summing the charge equivalents of exchangeable Na, K, Mg, Ca, Mn, Al, Fe, and H. Base saturation (BS) was calculated as the ratio of the sum of exchangeable Na, K, Mg, and Ca to the CEC. Concentrations of the elements in the extracts were determined by ICP-OES (Optima 3000, PerkinElmer Inc., Waltham, MA, United States). Soil particles were fractionated into sand, silt and clay using the pipette method according to Gee and Bauder (1986). Plant-available water storage capacity (AWC), i.e., the storable amount of water between field capacity and permanent wilting point, was calculated with a pedotransfer function according to Teepe et al. (2003) for each sampled soil layer for a thickness of 10 cm considering fine-earth density and soil texture, i.e., particle size distribution. Further details of the soil chemical and soil physical analysis are described in Frey et al. (2021).

In order to estimate fine-root biomass (<2 mm in diameter) and fine-earth densities, soil samples from the 1 L steel cylinders were sieved with a 4 mm sieve and the fine roots were sorted out (see also Frey et al., 2021). For soil microbial DNA sampling, fresh soil aliquots of about 5 g were put into a Ziploc^®^ bag and stored frozen at −80°C until DNA analyses. Finally, the remaining soil was dried at 105°C, and volumetric stone content and fine-earth density were determined.

To distinguish between topsoil and deep soil, data from soil layers L1 and L2 (topsoil; 0 – 25 cm) and from L5 and L6 (deepsoil; 110–155 cm) were summed and averaged. Soil measurements represent the mean for the topsoil and the deep soil, respectively. Data from L3, L4, and L7 were not included in this study.

### DNA Extraction and Shotgun Sequencing

DNA was extracted using the PowerSoil DNA Isolation Kit (Qiagen, Hilden, Germany) and was quantified using the high-sensitivity Qubit assay (Thermo Fisher Scientific, Reinach, Switzerland). Library preparation using the TruSeq DNA Library Prep Kit (Illumina Inc., San Diego, CA, United States) and shotgun sequencing of the eluted DNA samples was performed at Microsynth AG (Balgach, Switzerland), using the Illumina NovaSeq system (2 × 150 bp; Illumina Inc., San Diego, CA, United States). The twelve metagenomes were from three different forest sites (Chamoson, Neunkirch, and Saillon), each with two different forest types (beech forest and oak forest) and two different soil depths (topsoil and deepsoil). Extracted DNA was pooled from soil layers L1 and L2 for topsoil samples, and from layers L5 and L6 for deepsoil samples. Raw sequences were uploaded in the NCBI Sequence Read Archive under the accession number PRJNA783873.

### Assembly and Functional Annotation of Assembled Contigs

Pre-processing of metagenomic reads, assembly of reads into contigs, contig binning, and functional and phylogenetic annotation of contigs and bins were achieved using a customized pipeline (Donhauser et al., 2021; Perez-Mon et al., 2021). Briefly, raw reads were quality checked using FastQC v.0.11.9^1^. They were quality filtered and trimmed (i.e., pre-processed reads) using Trimmomatic v0.36 (*Q* = 20, minimum read length = 40; Bolger et al., 2014). Pre-processed read pairs and singleton reads from all samples were assembled into contigs (>200 bp) by iteratively building *de Bruijn* graphs using *k*-mers of increasing size with the *de novo* assembler MEGAHIT v1.1.3 (–*k*-min 27 –*k*-step 10; Li et al., 2015).

Genes were annotated to the coassembly (i.e., contigs obtained from the assembly of all the samples together) as this approach considerably increases the quality of the coding sequences (CDS) and gene annotation (e.g., Tamames et al., 2019). CDSs were predicted with MetaGeneMark v3.38 (Zhu et al., 2010). To uncover the potential metabolic capabilities of the soil metagenomes, CDS and genes were assigned to functions (i.e., functional genes). About 45% of the predicted genes (22,941,682) were assigned to general metabolic and cellular functions through EggNOG v4.5 (Evolutionary genealogy of genes: Non-supervised Orthologous Groups), which classifies the genes into clusters of orthologous groups (COGs) of proteins and organizes the COGs into general functional categories (Jensen et al., 2008; Huerta-Cepas et al., 2016). Annotation to EggNOG v4.5 was performed using the EggNOG-mapper v1.0.3 with the DIAMOND v.2.0.7 search mode against all protein sequences (Huerta-Cepas et al., 2017). About 1% of the protein-coding genes (507,956) were annotated to carbohydrate-active enzymes using the CAZy database (Carbohydrate-Active enZymes: release of July 2017 version; Cantarel et al., 2009). About 0.2% of the protein-coding genes (82,022) were annotated to N-cycling families using the N-cycling genes (NCyc) database (syn. NCycDB: curated integrative database for fast and accurate metagenomic profiling of NCyc clustered at 100% sequence identity; Tu et al., 2019). Annotations against the CAZy and NCyc databases were performed using SWORD v1.0.3 (Vaser et al., 2016) (−v 10^–5^; Anwar et al., 2019).

Kaiju v1.7.4, a program for sensitive taxonomic classification of high-throughput sequencing reads from metagenomic whole genome sequencing (Menzel et al., 2016), was used for the taxonomic classification of the assembled contigs using the prebuilt “nr + euk” database containing the bacterial, archaeal, viral, fungal, and microbial eukaryotic protein sequences from the NCBI BLAST non-redundant protein database (version 2021-02-24).

### Abundance Quantification of Protein-Coding Genes and Taxonomic Profiles

Pre-processed read pairs from each of the samples were mapped back to the assembled contigs, using the BWA aligner v0.7.15 (bwa-mem; Li, 2013). The function featureCounts from the package Subread v1.5.1 (-minOverlap 10, Q = 10, -primary, Liao et al., 2014) was used to count the reads mapped within the predicted protein coding. Taxonomy was also assigned by counting reads that mapped back to the taxonomic assigned contigs.

### Quantitative PCR of Functional Genes

Abundances of C- and NCyc were quantified using primers and thermocycling conditions as reported in Frey et al. (2020). Functional marker genes encoding for enzymes catalyzing major processes during methane oxidation (*pmoA*), methanogenesis (*mcrA*), denitrification (*nirS*, *nosZ*), nitrification (bacterial *amoA*, archaeal *amoA*, *nxrB*), and N fixation (*nifH*), are targeted. The specificity of the amplification products was confirmed by melting-curve analysis, and the expected sizes of the amplified fragments were checked in a 1.5% agarose gel stained with ethidium bromide. Three standard curves per target region (correlations ≥ 0.997) were obtained using tenfold serial dilutions (10^–1^ to 10^–9^ copies) of plasmids generated from cloned targets (Frey et al., 2011). Data was converted to represent average copy number of targets per μg DNA.

### Data Analyses

Statistical analyses were completed using the open-source software R v4.1.0 (R Core Team, 2019), and graphical representations of results were created with the R package *ggplot2* v3.3.5 (Wickham, 2016), unless specified otherwise. Protein-coding genes for which the sum of the reads over all soil samples was <10 were excluded from the analyses to lower the false discovery rate caused by stochasticity between samples.

To evaluate the dissimilarities in metabolic capabilities between the different soil habitats, we identified the functional genes - annotated against EggNOG, CAZy and NCyc databases – that were differentially abundant between the soils using the R package DESeq2 v 1.34 (Love et al., 2014). Data were normalized using DESeq2 and corrected for sequencing depth and annotated gene number variation, following the standard protocol used by the DESeq2 pipeline. All analyses were performed on the normalized dataset. To calculate the differentially abundant genes for each contrast, effect size shrinkage was applied using the lfcShrink() function with the ‘apeglm’ method (Zhu et al., 2019). Genes were reported as significantly over- or underrepresented only for pairwise comparisons with an adjusted *P*-value lower than 0.01; *P*-values were adjusted for multiple testing using the Benjamini–Hochberg method. Bray–Curtis dissimilarities between samples were visualized with non-metric multidimensional scaling (NMDS) (vegan R package; Oksanen et al., 2019). The Bray–Curtis dissimilarity matrix was calculated on the vst transformed gene dataset [varianceStabilizingTransformation() function from the DESeq2 package]. The envfit() function was then used to calculate and plot the environmental vectors in the NMDS orientation plot.

The statistical significance of observed differences was assessed with permutational analyses of variance (PERMANOVA, 10^5^ permutations, Monte Carlo approximated *p*-value). Multivariate homogeneity of group dispersions was checked prior to the PERMANOVAs to ensure that detected significant differences were associated with the tested factors and not with differences in the within-group variabilities. To calculate alpha diversity indices, the raw gene count dataset was resampled three times (*n* = 5,000,000) with the ‘rrarefy’ function from the R package ‘vegan.’ We calculated richness with the function ‘specnumber,’ and the Shannon index with the diversity() function.

## Results

### Forest Site Property Change With Depth

Analysis of soil chemical and physical parameters at the six forest sites showed that they were not dependent on the dominant tree genus at the site, as there were no significant differences in any of these variables. In contrast, some of the soil properties showed significant changes with soil depth (Table 1). Soil pH increased significantly with soil depth, whereas soil base saturation overall was high, around 100%. Both, *C*_org_ and *N*_tot_ decreased significantly with soil depth, *C*_org_ about tenfold and *N*_tot_ about fourfold. In contrast, soil density increased significantly with soil depth by about a factor of 1.5 (Table 1).

**TABLE 1:** Mean values of soil biological, chemical, and physical properties (*n* = 3 forest sites).

	Beech sites		Oak sites		*p*-ANOVA^+^		
Soil layer type^#^:	Topsoil	Deepsoil	Topsoil	Deepsoil	Tree	Depth	Interaction
**Soil biological properties:**							
Microbial biomass (μg DNA g^–1^ soil)*	16.7	1.73	10.4	1.44	0.06	**<0.001**	0.08
Fine-root biomass (g dm^–3^ soil)	2.53	0.29	3.28	0.31	0.06	**<0.001**	0.07
**Soil chemical properties:**							
pH (CaCl_2_)	6.73	7.67	6.83	7.70	0.85	**0.029**	0.92
Base saturation (%)	99	99.7	100	99.9	0.53	0.20	0.44
*C*_org_ (%)	5.15	0.74	4.34	0.48	0.66	**0.006**	0.83
*N*_tot_ (%)	0.36	0.08	0.30	0.08	0.66	**0.015**	0.72
*C*:*N* ratio	13.8	13.6	14.2	8.2	0.48	0.39	0.42
**Soil physical properties:**							
Fine-earth density (g cm^–3^)	0.73	1.12	0.81	1.11	0.60	**<0.001**	0.46
Stone content (vol %)	15.8	43.3	16.7	47.5	0.88	0.11	0.92
Sand (%)	17.2	33.3	19.8	43.4	0.14	0.06	0.86
Silt (%)	46.7	46.6	38.7	43.7	0.62	0.82	0.82
Clay (%)	36.3	16.0	31.7	13.0	0.68	0.06	0.93
AWC_10_ (mm)^‡^	22.4	13.3	33.6	11.9	0.49	0.05	0.38

*^+^Effects of tree genus, soil depth, and their interaction were assessed by analysis of variance (ANOVA); significant values (p < 0.05) are in bold.*

*^#^Soil layer types: topsoil: mean of L1 (0–10 cm) + L2 (15–25 cm); deepsoil: mean of L5 (110–125 cm) + L6 (140–155 cm) (for details see Frey et al., 2021).*

*Soil DNA content as a proxy for soil microbial biomass.

*^‡^Plant-available water storage capacity (AWC) per 10 cm soil thickness.*

Analysis of fine-root variables indicated, that oak sites had no significantly greater fine-root biomass compared with beech sites (Table 1). However, the fine-root biomass decreased significantly with increasing soil depth in both tree species by about tenfold, similarly to the microbial biomass, which also decreased significantly by about tenfold (Table 1).

### Overall Metagenome Sequencing Results

After quality filtering of the metagenomes of twelve soil samples we obtained in total 1.6 × 10^9^ high-quality reads (ranging from 1.2 to 1.7 × 10^8^ reads per soil sample). The assembly consisted of 3.7 × 10^7^ MEGAHIT contigs of 568 bp on average, ranging from 202 bp to 4.1 × 10^5^ bp, with a *N*_50_ value of 581 bp and a GC-content of 64% (Supplementary Table 2). We found a significantly higher percentage of reads mapped to contigs and to protein coding genes in the deep soil (contigs: 83.3%; CDS: 66.2%) than in the topsoil (contigs: 71.7%; CDS: 53.6%) (Table 2), whereas the percentages did not differ significantly between the tree genera. In total, 5.0 × 10^7^ predicted genes were found among the contigs, 2.3 × 10^7^ of which we could annotate with the EggNOG database, 5.1 × 10^5^ with the C-cycling gene database CAZy, and 8.2 × 10^4^ with the N-cycling gene database NCyc (Supplementary Table 2). The fraction of the predicted genes annotated by each database were 45.1% (for EggNOG), 1.0% (for CAZy), and 0.2% (for NCyc).

**TABLE 2:** Mean number of sequences and percentage of protein-coding genes (CDS genes), and the relative abundance of contigs assigned to taxa at the domain level for topsoil or deepsoil (*n* = 3 forest sites).

	Beech sites		Oak sites		*P*-ANOVA^+^		
	Topsoil	Deepsoil	Topsoil	Deepsoil	Tree	Depth	Interaction
Raw reads (×10^8^)	1.33	1.54	1.41	1.33	0.43	0.47	0.13
High-quality reads (×10^8^)	1.28	1.44	1.35	1.27	0.48	0.50	0.13
Reads mapped to contigs (%)	71.5	83.5	71.8	83.1	0.98	**<0.001**	0.85
Reads mapped to CDS genes (%)	53.8	66.2	53.3	66.3	0.94	**0.001**	0.93
Archaea (%)	0.32	1.61	0.22	2.04	0.80	**0.034**	0.67
Bacteria (%)	91.83	91.17	92.10	91.47	0.65	0.31	0.98
Eukarya (%)	0.44	0.37	0.45	0.34	0.86	**0.035**	0.54
Viruses (%)	0.06	0.05	0.05	0.03	0.23	0.44	0.94
Unclassified (%)	7.32	6.80	7.16	6.13	0.45	0.17	0.64

*^+^Effects of tree genus, soil depth, and their interaction were assessed by analysis of variance (ANOVA); significant values (P < 0.05) are in bold.*

### Taxonomic Composition of the Metagenomes

Contigs were classified to different taxa, whereas reads mapped to classified contigs were used to quantify the taxa abundance. Bacteria were always the most common group in all samples, with relative abundances of >90% in each sample, whereby soil depth had no significant influence on abundance. Archaea were 5–9 times more abundant in the deep soil than in the topsoil, whereas both Bacteria and Archaea were unaffected by the tree genus (Table 2). In contrast to Archaea, Eukarya were more abundant in the topsoil than in the deep soil (Table 2). Viruses were always the least abundant group and did not differ between soil depths or tree genera (Table 2).

The most abundant archaeal phyla, such as Crenarchaeota, Euryarchaeota, and Thaumarchaeota, tended to be significantly more abundant in the deep soil than in the topsoil (Table 3). In particular, the classes of the anaerobic Methanomicrobia and the chemoautotrophic nitrifying Nitrososphaeria were significantly more abundant in the deep soil. Among the Bacteria, the phyla Actinobacteria, Chloroflexi, and Nitrospirae were significantly more abundant in the deep soil, whereas Proteobacteria were more abundant in the topsoil (Table 3). Among them the bacterial classes such as Actinomycetia, Anaerolineae, Chloroflexia, and Nitrospira were significantly more abundant in the deep soil, whereas the classes Alphaproteobacteria and Gammaproteobacteria were significantly more abundant in the topsoil.

**TABLE 3:** Mean number of reads assigned to taxonomic contigs of selected phyla and classes of the domains Archaea and Bacteria (*n* = 3 forest sites).

		Beech sites		Oak sites		*P*-ANOVA^+^		
Phylum	Class	Topsoil	Deepsoil	Topsoil	Deepsoil	Tree	Depth	Interaction
**Archaea:**								
Crenarchaeota (×10^6^)		0.03	0.06	0.02	0.06	0.63	**0.001**	0.99
	Thermoprotei (×10^6^)	0.02	0.05	0.02	0.05	0.98	**0.001**	0.80
Euryarchaeota (×10^6^)		0.42	4.02	0.38	6.37	0.64	0.08	0.62
	Methanomicrobia (×10^6^)	0.15	0.22	0.13	0.22	0.62	**0.001**	0.62
	Thermoplasmata (×10^6^)	0.03	0.08	0.03	0.12	0.54	0.08	0.51
Thaumarchaeota (×10^6^)		0.33	0.93	0.05	0.59	0.19	**0.029**	0.87
	Nitrososphaeria (×10^6^)	0.03	0.29	0.01	0.20	0.47	**0.010**	0.63
**Bacteria:**								
Actinobacteria (×10^6^)		34.98	62.34	47.33	87.74	0.12	**0.015**	0.57
	Actinomycetia (×10^6^)	30.41	52.59	40.94	74.87	0.09	**0.011**	0.51
Chloroflexi (×10^6^)		5.66	30.11	6.41	27.34	0.83	**0.001**	0.71
	Anaerolineae (×10^6^)	0.40	1.35	0.37	0.81	0.22	**0.012**	0.27
	Chloroflexia (×10^6^)	0.27	0.56	0.29	0.56	0.93	**0.002**	0.92
Nitrospinae (×10^6^)		0.26	0.24	0.29	0.21	0.94	0.39	0.53
	Nitrospinia (×10^6^)	0.03	0.03	0.04	0.03	0.73	0.65	0.26
Nitrospirae (×10^6^)		1.45	3.82	1.01	2.46	0.06	**0.002**	0.29
	Nitrospira (×10^6^)	0.80	1.29	0.55	0.90	**0.046**	**0.015**	0.59
Proteobacteria (×10^6^)		120.04	71.69	113.89	74.08	0.71	**<0.001**	0.41
	Alphaproteobacteria (×10^6^)	58.76	23.54	62.95	26.70	0.46	**<0.001**	0.92
	Betaproteobacteria (×10^6^)	28.39	22.36	21.50	23.68	**0.029**	0.10	**0.004**
	Deltaproteobacteria (×10^6^)	15.41	13.38	14.11	11.77	0.18	0.06	0.88
	Gammaproteobacteria (×10^6^)	13.83	9.85	12.40	9.58	0.48	**0.018**	0.26

*^+^Effects of tree genus, soil depth, and their interaction were assessed by analysis of variance (ANOVA); significant values (P < 0.05) are in bold.*

### Diversity of the Protein-Coding Genes Changes With Soil Depth

There was an overall significant effect of the soil depth on the alpha-diversity of functional genes calculated over the entire dataset (Pseudo-*F* = 17.7, *R*^2^ = 0.34, *P* < 0.001 for richness, Pseudo-*F* = 17.2, *R*^2^ = 0.34, *P* < 0.001 for Shannon index), as well as for the CAZy dataset (Pseudo-*F* = 6.33, *R*^2^ = 0.16, *P* = 0.016 for richness, Pseudo-*F* = 13.9, *R*^2^ = 0.29, *P* < 0.001 for Shannon index) and the NCyc dataset (Pseudo-*F* = 12.7, *R*^2^ = 0.27, *P* < 0.001 for Shannon index; Supplementary Table 3). The tree genus, in contrast, did not show any significant effect on the alpha-diversity for any of the three datasets. In the analysis of the entire dataset, however, site had a weak significant effect on Shannon index (Pseudo-*F* = 3.30, *R*^2^ = 0.17, *P* = 0.048) but not on richness (Supplementary Table 3). This significance was mainly attributed to the fact that the Shannon indices at the site Neunkirch site varied only marginally, whereas at Chamoson and Saillon they varied rather strongly (Supplementary Figure 1).

### Soil Depth but Not Tree Genus Change the Functional Gene Structure

**FIGURE 1:**
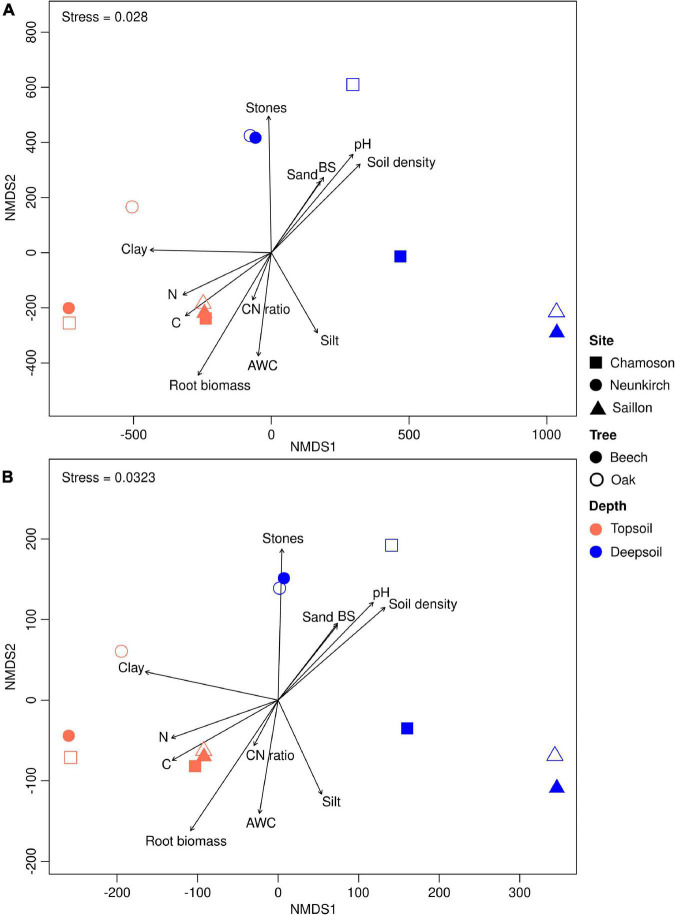
Non-metric multidimensional scaling (NMDS) ordination based a Euclidean distance matrix calculated on the variance stabilizing transformation (VST) transformed **(A)** CAZy gene and **(B)** NCyc gene dataset. Abiotic environmental variables were projected as arrow vectors onto the NMDS ordinations. Plant-available water storage capacity (AWC) per 10 cm soil thickness; BS, base saturation (see also Frey et al., 2021).

For the functional genes annotated with C- and NCyc, topsoil was clearly separated from deep soil. The NMDS ordination for the CAZy and NCyc gene datasets in topsoil and deep soil of beech and oak sites indicated a strong separation of the functional genes (stress = 0.028 and 0.032, respectively; Figure 1). We further identified the individual soil environmental variables explaining the functional gene structures. Correlations of the genes with soil environmental variables revealed that fine-root biomass, clay content, and plant-available water storage capacity (AWC) were related in the topsoil, whereas in the deep soil the genes correlated closely with pH, base saturation (BS), and fine-earth density.

The functional gene structure of all predicted genes showed a clear separation between the metagenomes of the topsoil and the deepsoil. This was confirmed by the PERMANOVA, where soil depth explained most of the variance and was a main factor driving sample clustering (Pseudo-*F* = 5.64, *R*^2^ = 0.36, *P* = 0.003; Table 4). In contrast, site and tree genus were not significant explanatory factors. Similarly, for the CAZy and NCyc datasets, the functional gene structure of the metagenomes in the deep soil was significantly different from that in the topsoil (CAZy genes: Pseudo-*F* = 4.89, *R*^2^ = 0.33, *P* = 0.002, NCyc genes: Pseudo-*F* = 5.38, *R*^2^ = 0.35, *P* = 0.002; Table 4).

**TABLE 4:** Results of a PERMANOVA testing the effects of various factors for all predicted genes, for the CAZy genes, and for the NCyc genes.

	Factor	*DF*	Pseudo-*F*	*R* ^2^	*P*
All genes	Site	2	1.38	0.23	0.18
	Tree	1	0.46	0.04	0.87
	Depth	1	5.64	0.36	**0.003**
CAZy genes	Site	2	1.44	0.24	0.15
	Tree	1	0.51	0.05	0.85
	Depth	1	4.89	0.33	**0.002**
NCyc genes	Site	2	1.38	0.23	0.17
	Tree	1	0.50	0.05	0.85
	Depth	1	5.38	0.35	**0.002**

*Values represent degrees of freedom (DF), F-value (Pseudo-F), strength of the correlation (R^2^), and the level of significance (P); significant values (P < 0.05) are in bold.*

### Differentially Abundant Genes in Deep Soils

**FIGURE 2:**
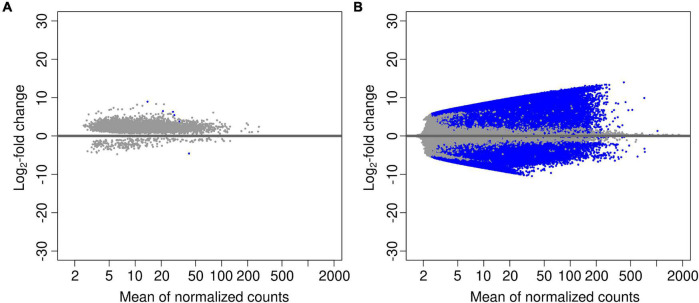
Volcano plots performed on pairwise comparisons for the factors **(A)** “tree” beech *vs.* oak, and **(B)** “soil depth” deepsoil vs. topsoil. Blue data points represent the genes that are differentially abundant (adjusted *P*-value < 0.01).

In order to investigate changes in the abundance of functional genes with site, tree genus and soil depth, we calculated log_2_-fold changes in all predicted genes, and for the genes annotated with the CAZy and NCyc databases (Table 5). The comparison of all genes between deepsoil and topsoil agglomerated over the three forest sites resulted in 5.4 × 10^5^ over- and 3.1 × 10^5^ underrepresented genes (*P* < 0.01; Table 5 and Figure 2). Pairwise comparisons of the three forest sites indicated that there were about 10-times fewer over- or underrepresented genes than found in the comparison between topsoil and deepsoil (overrepresented: 9–25 × 10^3^; underrepresented: 9–75 × 10^3^; Table 5). The genes annotated to the CAZy and the NCyc databases showed the most over- and underrepresented genes in the comparison of the topsoil with the deep soil. Thus, most of the differentially abundant genes explained most of the differences between deepsoil and topsoil samples, with the majority of genes were overrepresented in deep soil samples. In contrast, comparisons of the two tree genera indicated <10 over- or underrepresented genes when considering all genes, as well as for the genes annotated with the CAZy and NCyc databases (Table 5).

**TABLE 5:** Pairwise comparison and number of significantly (*P* < 0.01) over- and underrepresented differentially abundant genes out of all predicted genes, of the CAZy genes, and of the NCyc genes.

		All genes		CAZy genes		NCyc genes	
Factor	Pairwise comparison	Overrep.	Underrep.	Overrep.	Underrep.	Overrep.	Underep.
Site	Saillon vs. Chamoson	9,030	34,894	447	996	42	124
	Neunkirch vs. Saillon	23,598	74,901	1,399	3,229	142	395
	Neunkirch vs. Chamoson	25,410	9,030	1,117	446	135	37
Tree	Beech vs. oak	8	1	1	0	0	0
Depth	Deepsoil vs. topsoil	544,454	305,491	17,723	10,135	2,655	1,171

### Change in the Abundance of EggNOG, CAZy and NCyc Superfamilies With Depth

Functional genes annotated against EggNOG database were used to assess overall changes in functional genes in response to soil depth and tree genus. The most abundant categories were amino acid transport (E), energy production and conversion (C), signal transduction (T), and replication, recombination, and repair (L). The first three categories had significantly more abundant genes in the deep soil than in the topsoil, whereas soil depth had no effect on the abundance of genes in the L category (Supplementary Table 4). Aggregating over all three levels of functional categories, the categories that increased most significantly in the deep soil were on the levels of “cellular processes and signaling genes” (7 of 10 categories) and “metabolism genes” (5 of 8 categories; Supplementary Table 4). The only category with genes that significantly less abundant in the deep soil was cell motility (N). In contrast, tree genus did not have any significant influence on the number of genes of any category.

Overall, the most abundant genes annotated to the CAZy database were found in the category of glycoside transferases (GT) and glycoside hydrolases (GH), followed by carbohydrate binding modules (CBM). In particular, GT and CBM were significantly more abundant in the deep soil than in the topsoil (Table 6). This indicates, that genes involved in the degradation of cellulose and chitin (CBM) were overrepresented in the deep soil, as well as genes for anabolic processes to build new tissues (GT), which play essential roles in biosynthesis pathways of oligo- and polysaccharides, as well as protein glycosylation and the formation of valuable natural products.

**TABLE 6:** Number of CAZy and NCyc genes (*n* = 3 forest sites).

	Beech sites		Oak sites		*P*-ANOVA^+^		
	Topsoil	Deepsoil	Topsoil	Deepsoil	Tree	Depth	Interaction
**CAZy genes:**							
Auxiliary activities (AA) (×10^6^)	0.25	0.27	0.23	0.31	0.71	0.07	0.28
Carbohydrate binding (CBM) (×10^6^)	1.38	2.05	1.19	2.20	0.95	**0.017**	0.36
Carbohydrate esterases (CE) (×10^6^)	0.50	0.55	0.45	0.58	0.84	0.12	0.46
Glycoside hydrolases (GH) (×10^6^)	2.50	2.65	2.30	3.00	0.86	0.21	0.42
Glycosyl transferases (GT) (×10^6^)	2.81	3.64	2.59	3.89	0.97	**0.015**	0.52
Polysaccharide lyases (PL) (×10^6^)	0.10	0.13	0.09	0.14	0.96	0.06	0.49
**NCyc genes:**							
Anammox (Ana) (×10^6^)	0.0007	0.0006	0.0009	0.0006	0.62	0.32	0.65
Assimilatory nitrate red. (Anr) (×10^6^)	0.11	0.11	0.10	0.12	0.99	0.10	0.14
Denitrification (Den) (×10^6^)	0.06	0.06	0.05	0.06	0.29	0.19	0.36
Dissimilatory nitrate red. (Dnr) (×10^6^)	0.05	0.05	0.05	0.06	0.89	0.37	0.25
Nitrification (Nit) (×10^6^)	0.0031	0.0035	0.0010	0.0024	0.09	0.32	0.58
Nitrogen fixation (Nif) (×10^6^)	0.00001	0.00001	0.00000	0.00003	0.13	**0.039**	**0.031**
Organic degradation/synthesis (×10^6^)	0.75	0.98	0.68	1.07	0.86	**0.005**	0.37
Others (×10^6^)	0.0021	0.0013	0.0084	0.0013	0.15	0.65	0.17

*^+^Effects of tree genus, soil depth, and their interaction were assessed by analysis of variance (ANOVA), significant values (P < 0.05) are in bold.*

The most abundant of N-cycling families were those involved in the organic degradation and synthesis (Ods), followed by assimilatory nitrate reduction (Anr), denitrification (Den), and dissimilatory nitrate reduction (Dnr). The family Ods was significantly more abundant in the deep soil, whereas the Anr family only tended to increase in the deep soil (Table 6). Although genes associated to nitrogen fixation (Nif) were significantly abundant in the deep soil, the number of genes was very low overall in the forest soils. No N-cycling families were affected by the tree genus (Table 6).

### Functional Categories of Differentially Abundant Genes Annotated to EggNOG, CAZy, and NCyc

In order to investigate changes in the abundance of functional genes with soil depth, we calculated log_2_-fold changes (LFC) for the genes annotated with EggNOG. Significantly differently abundant microbial functional genes (>1 or <1 LFC) with greater numbers of genes in the deep soil were observed in all functional categories, with the highest values for nuclear structure, translation, and cell cycle control (Supplementary Figure 2). Furthermore, we were particularly interested in the changes in the abundance of functional genes involved in carbohydrate transport and metabolism (Supplementary Figure 3) and amino acid transport and metabolism (Supplementary Figure 4). Although not significant as a whole functional category, a majority of the genes associated with carbohydrate transport and metabolisms were highly significantly more abundant in the deep soil, with the highest positive LFC values corresponding to the genes for glucanotransferase (COG1640), sugar and pyruvate kinases (COG2379, COG0469, COG1080), hydrolase of glycosyl (COG1543), dehydrogenase of phosphogluconate (COG1023), citrate lyase (COG2301), and mannosidase (COG0383; Supplementary Figure 3). The large majority of the genes of the category amino acid transport and metabolism were significantly enhanced in the deep soil, with highest positive LFC of the genes for ABC-type amino acid transporters (COG4177), aminopeptidases (COG2234), amidohydrolases (COG1228), dehydrogenases of alanine and aspartate (COG0686, COG0136), and synthetases (COG0128, COG0337; Supplementary Figure 4).

**FIGURE 3:**
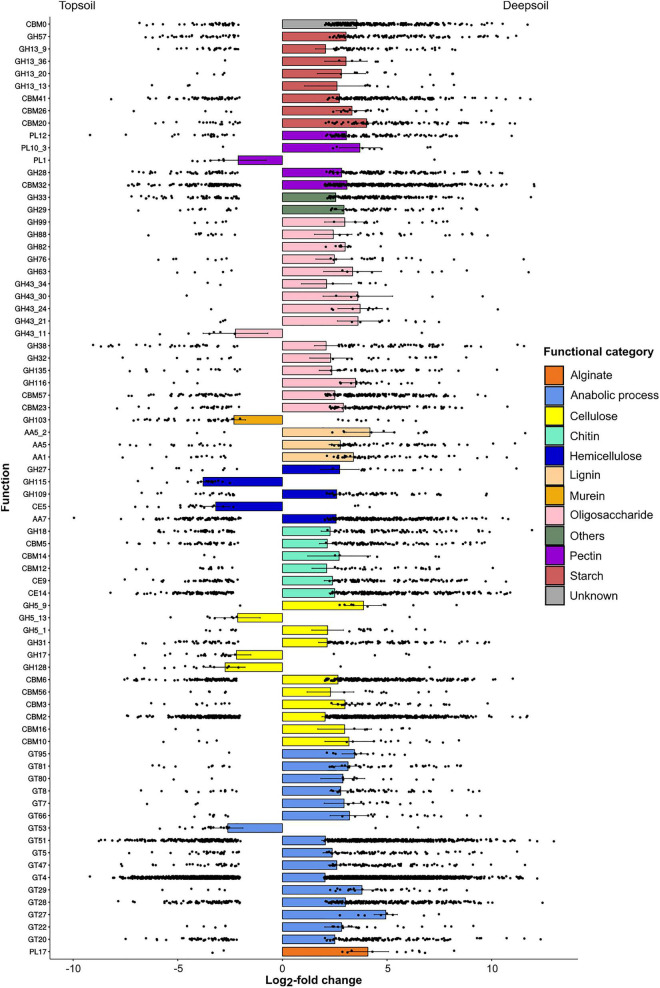
Under- and overrepresented genes annotated to the CAZy database for the pairwise comparison of topsoil vs. deepsoil. Only significantly (*P* < 0.01) differentially abundant genes between the two soil depths whose log_2_-fold change was lower than –2 or higher than +2 are displayed.

The total number of differentially abundant genes annotated to CAZy 35,493 (6.99% of the total number of genes), and the abundance of most of these genes differed between soil depths. The comparison between topsoil and deepsoil indicated 10,135 under- and 17,723 overrepresented differentially abundant genes (Table 5). In the family of glycosyl hydrolases (GH family), the genes with the highest positive LFC in the deep soil were involved in the activity of alpha-amylase (GH13 and GH57; involved in starch degradation), alpha-L-rhamnosidase (GH28; pectin), beta-xylosidase (GH43 and GH116; oligosaccharides), 2-mannosidase (GH99; oligosaccharides), alpha-galactosidase (GH27; hemicellulose), alpha-*N*-acetylgalactosaminidase (GH109; hemicellulose), chitinase (GH18, chitin), and endo-1-4-glucanase (GH5, cellulose) (Figure 3). In the family of the auxiliary activities (AA family), the main activities favored in the deep soil were laccase (AA1; lignin), manganese peroxidase (AA5_2; lignin), and oxidase (AA7; lignin). Genes of the family of carbohydrate binding modules family (CBM family) were also favored in the deep soil, and these genes were mainly involved in the binding of starch (CBM20, CBM26, and CBM41), pectin (CBM32), chitin (CBM12 and CMB14), and cellulose (CBM2, CBM3, CBM6, CBM10, CBM16, and CBM56). In the polysaccharide lyase family (PL family), the genes with the highest positive LFC in the deep soil were pectate lyase (PL10_3; pectin) and heparin lyase (PL12; pectin). Genes of the glycosyl transferase family (GT family) were significantly more abundant in the deep soil, and these genes were mainly involved in the catalyzation of polypeptide transferase (GT27), sialyltransferase (GT29), or arabinofuranosyltransferase (GT95), or in the *N*-glycosylation of proteins (GT66). In contrast, some genes had a significantly higher abundance in the topsoil, and these genes were mainly involved in the degradation of pectin (PL1: pectin lyase), oligosaccharides (GH43_11; xylan 1,4-β-xylosidase), murein (GH103, peptidoglycan lytic transglycosylase), hemicellulose (GH115; xylan α-1,2-glucuronidase), cellulose (GH5_13; GH17, GH128; cellulase) and cutin (CE5; cutinase).

**FIGURE 4:**
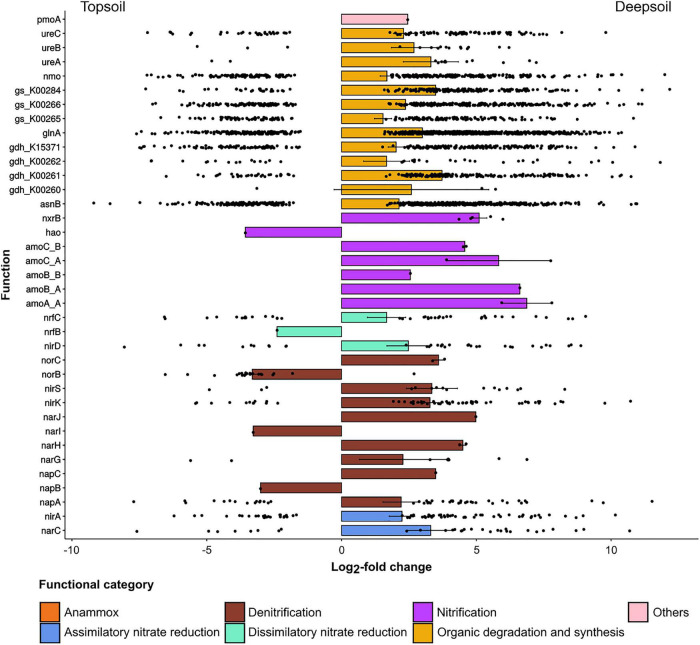
Under- and overrepresented genes annotated to the NCyc database for the pairwise comparison of topsoil vs. deepsoil. Only significantly (*P* < 0.01) differentially abundant genes between the two soil depths whose log_2_-fold change was lower than –1.5 or higher than +1.5 are displayed.

For NCyc, the total number of differentially abundant genes was 4,701 (the 5.87% of the total number of genes), and most of these genes varied with soil depth. The comparison between topsoil and deepsoil indicated 1,171 under- and 2,655 overrepresented differentially abundant genes (Table 5). In the comparisons between deepsoil and topsoil, the family of organic degradation and synthesis genes (Ods: ammonification, assimilation) was overrepresented (positive LFC) in the deep soil, with the LFC highest in the deep soil corresponding to the genes *ureB, ureA*, *gs_K00284, glnA* and *gdh_K00261* (Figure 4). In the family of nitrification genes (Nit), the genes *nxrB*, *amoA_A, amoB_A, amoC_A, amoB_B*, and *amoC_B* had higher LFC values in the deep soil. In the denitrification (Den) and dissimilatory nitrate reduction (Dnr) gene families, *norC, nirS, nirK, nirD, narJ, narH*, and *napC* were overrepresented in the deep soil. Furthermore, *nirA* and *narC* in the family of assimilatory nitrate reductase (Anr) and the gene *pmoA* (ammonia/methane oxidation) were more abundant in the deep soil (Figure 4). In contrast, only a few genes were overrepresented in the topsoil, i.e., *nrfB, norB, narl, and napB*, as well as *hao* (Figure 4).

**FIGURE 5:**
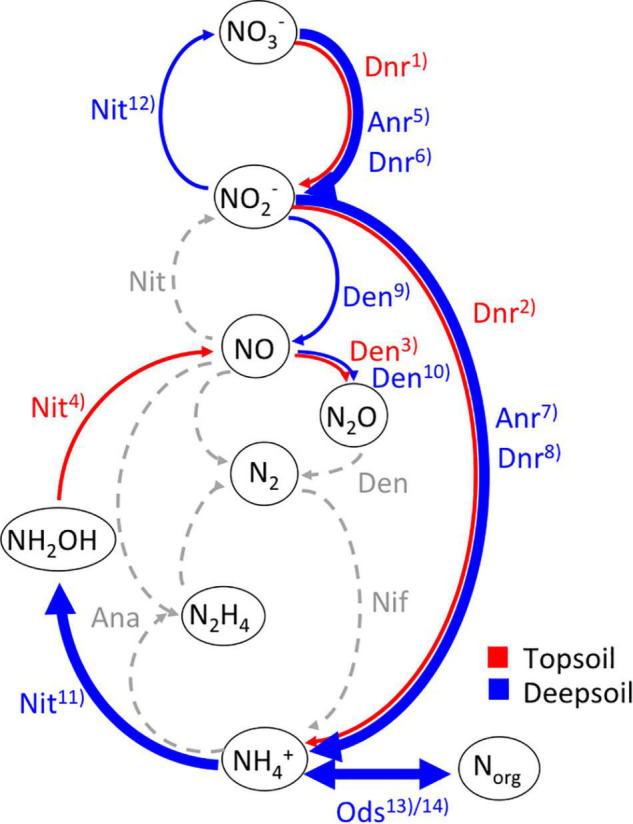
Summary of microbial transformations of N compounds in (red) topsoil and (blue) deepsoil according to the significantly differentially abundant genes between topsoil and deepsoil annotated to the NCyc database following the scheme of Kuypers et al. (2018). Dashed arrows show the hypothetical direction without any differentially abundant genes, thin arrows have one or two increased differentially abundant genes, bold arrows have more than two increased differentially abundant genes between the two soil depths. Nitrogen-transforming processes: Ana, anammox; Anr, assimilatory nitrate reduction; Den, denitrification; Dnr, dissimilatory nitrate reduction; Nit, nitrification; Nif, nitrogen fixation; Ods, organic degradation and synthesis (^13)^ammonification, ^14)^assimilation). Enzymes performing N transformations according to Tu et al. (2019): (1) *napB*, *narI*; (2) *nrfB*; (3) *norB*; (4) *hao*; (5) *narC;* (6) *napA, napC*, *narG, narH, narJ*; (7) *nirA*; (8) *nirD, nrfC*; (9) *nirK*, *nirS*; (10) *norC*; (11) *amoA*_A, *amoB*_A, *amoC*_A, *amoB*_B, *amoC*_B; (12) *nxrB*; (13) *gdh*_K00260; *gdh*_K00261, *gdh*_K00262, *gdh*_K15371, *nmo*, *ureA, ureB, ureC*; (14) *asnB, glnA, gs*_K00265, *gs*_K00266, *gs*_K00284.

A summary of the differentially abundant genes annotated to the NCyc database for topsoil and deep soil is shown in Figure 5. It is apparent that mainly the pathways for nitrification (Nit), assimilatory and dissimilatory nitrate reduction (Anr and Dnr), and organic degradation and synthesis (Ods) are increased in the deep soil (Figure 5).

### Quantitative PCR of C- and N-Transforming Processes

Gene abundances of C- and N-transforming processes were mainly driven by soil depth and not the tree genus. Genes involved in nitrification (archaeal *amoA*: *P* = 0.039 and *nxrB*: *P* < 0.001) were considerably more abundant in the deep soil compared with the topsoil (Table 7). In contrast, genes involved in denitrification (*nosZ*: *P* = 0.002), methane oxidation (*pmoA*: *P* = 0.039) and methanogenesis (*mrcA*: *P* = 0.002) were significantly less abundant in the deep soil than in the topsoil (Table 7).

**TABLE 7:** Mean values of gene abundances of C- and N-transforming processes, and assessed by quantitative PCR (*n* = 3 forest sites).

		Beech sites		Oak sites		*P*-ANOVA^+^		
Process	Gene^#^	Topsoil	Deepsoil	Topsoil	Deepsoil	Tree	Depth	Interaction
**C-transforming process:**								
Methane oxidation (×10^6^)	*pmoA*	4.25*	3.20	4.57	3.06	0.86	**0.039**	0.67
Methanogenesis (×10^6^)	*mrcA*	0.05	0.02	0.07	0.02	0.39	**0.002**	0.52
**N-transforming processes:**								
Denitrification (Den) (×10^6^)	*nirS*	27.53	48.49	31.31	20.73	0.47	0.75	0.35
	*nosZ*	16.01	7.40	20.73	6.45	0.46	**0.002**	0.28
Nitrification (Nit) (×10^6^)	*amoA_*Arch.	18.10	43.20	1.21	24.86	0.11	**0.039**	0.94
	*amoA*_Bact.	0.67	0.40	0.37	0.27	0.39	0.46	0.73
	*nxrB*	21.56	85.51	5.59	70.89	0.20	<**0.001**	0.95
Nitrogen fixation (Nif) (×10^6^)	*nifH*	9.21	3.84	4.51	2.29	0.23	0.15	0.53

*^+^Effects of tree genus, soil depth, and their interaction were assessed by analysis of variance (ANOVA), significant values (P < 0.05) are in bold.*

*^#^Genes encoding for enzymes in the C and N transforming processes: amo, ammonia monooxygenase; mrc, methyl coenzyme M (methyl-CoM) reductase; nif, nitrogenase; nir, nitrite reductase; nos, nitrous oxide reductase; nxr, nitrite oxidoreductase; pmo, particulate methane monooxygenase.*

*Gene copy numbers are given in μg^–1^ DNA.

## Discussion

### Altered Microbial Communities in Deep Soil Layers

In deep soil layers, the relative abundance of contigs assigned to Archaea was 5–9 times higher than in the topsoil, while the relative abundance of contigs assigned to Bacteria was not influenced by soil depth. Similarly, both Bacteria and Archaea were unaffected by the tree genus. In deep soil layers, the classes of the chemoautotrophic nitrifying Nitrososphaeria of the archaeal phylum Thaumarchaeota and the anaerobic Methanomicrobia of the archaeal phylum Euryarchaeota were particularly abundant. Archaea have previously been regarded as rare microorganisms that play a limited role in biogeochemical cycles in non-extreme environments (Moissl-Eichinger et al., 2018). Recently, however, Archaea have been found to be distributed widely and comprise a broad diversity of gene repertoires and lifestyles (Martínez-Núñez and Pérez-Rueda, 2016). These authors suggested that Archaea contain more promiscuous enzymes, i.e., the ability of an enzyme to catalyze a random side reaction in addition to its main reaction, providing them with an enzymatic repertoire that enables them to face multiple ecological challenges in harsher environments such as the deep soil.

It is not unexpected to find Nitrososphaera, a class within the phylum Thaumarchaeota, in deep soils. Thaumarchaeota are generally negatively correlated with clay and *C*_org_. Our deep soil layers are characterized by a high sand and low *C*_org._ content, indicating well-drained and oligotrophic conditions. However, ecological niche preferences for ammonia-oxidizing Archaea (AOA) are only found in the literature for topsoil (e.g., Saghaï et al., 2021) and data from deep soils are not available as they are currently very poorly explored. Among the Bacteria, the phyla Nitrospirae, Chloroflexi, and Actinobacteria were significantly more abundant in deep soil. In general, our shotgun metagenomics data are consistent with the results of Frey et al. (2021), who used the same forest sites as in the present study using amplicon sequencing of 16S rRNA genes.

The increased occurrence of some groups of less-known prokaryotes, e.g., Archaea, Chloroflexi, or Nitrospirae at greater soil depths indicates that living conditions for microorganisms change to an oligotrophic and a more oxygen limited environment in deep soil. Such organisms gain their energy from the oxidation of inorganic atoms or molecules as a growth-supporting reductant such as ammonia or nitrite (Hooper and Dispirito, 2013; Kelly and Wood, 2013). Taxa of Thaumarchaeota couple ammonia oxidation with C fixation (Könneke et al., 2014), and taxa of Nitrospirae are able to oxidize nitrite to nitrate or even facilitate the complete cycle from ammonia to nitrite to nitrate (commamox; Daims et al., 2015). Moreover, AOA are favored by oligotrophic soil conditions (Ollivier et al., 2011). Taxa of Methanomicrobia are able to produce methane by using CO_2_ (Enzmann et al., 2018), and taxa of Actinobacteria form one of the few bacterial groups able to degrade lignin (Abdel-Hamid et al., 2013). However, it also indicates that deep soil microorganisms have to invest more effort into the mobilization of C and N than microorganisms in the topsoil, because they gain their energy from the oxidation of inorganic compounds (lithotrophs), which has a higher cost than required to gain energy from the oxidation of organic compounds (organotrophs) (Hooper and Dispirito, 2013).

### Functional Gene Structure and Differentially Abundant Genes

Using shotgun metagenomics, we primarily investigated the potential of functional profiles. The functional gene structure of all predicted genes showed a clear separation between the metagenomes of the topsoil and the deepsoil, and similarly, for the EggNOG, CAZy, and NCyc datasets. Moreover, most of the differentially abundant genes contributed to the difference in functional gene structure observed between the deep soil and the topsoil, with the majority of genes being overrepresented in the deep soil samples (>5 × 10^5^ genes). In contrast, the comparison of the two tree genera beech and oak showed only a few over- or underrepresented genes when all genes were considered, as well as for the genes annotated with the above-mentioned databases. The three forest sites also differed in functional gene structure when compared to each other, but the differentially abundant genes were more than ten times less abundant than when comparing the two soil depths. Therefore, we concluded that the comparison between the topsoil and the deepsoil is the most relevant.

Functional genes annotated against the EggNOG database, which maps predicted genes of general metabolic and cellular functions, showed that the majority of the functional gene categories were significantly overrepresented in the deep soil. In particular, genes of the functional categories such as cell cycle control, nucleotide transport, or amino acid transport and metabolism were highly significantly more abundant. This overall could indicate that in the deep soil altered cellular and metabolic processes occur in the deep soil, which affect the growth of cells and the mobilization of nutrients. Among the most overrepresented genes of the carbohydrate and amino acid transport and metabolism categories were ABC-type amino acid transporters, peptidases, synthetases, transferases, kinases, hydrolases, dehydrogenases, lyases, and mannosidases. Such a dominance of overrepresented functional gene categories is surprising but not unusual, as Perez-Mon et al. (2021) found a similar pattern when comparing permafrost soils with active-layer soils in a high mountain soil in the Swiss Alps. Likewise, in a comparison of cultivated and uncultivated chernozems in Russia, the majority of the functional gene categories were overrepresented in the virgin soil (Gorbacheva et al., 2018). Similarly, Chen et al. (2021) reported, by using the KEGG (Kyoto Encyclopedia of Genes and Genomes) database, that microorganisms in bare soils, compared with those in vegetated soils, have greater relative abundances of genes associated with metabolic functions. They concluded that bare soils harbor a higher proportion of genes that are not available in public reference databases. This is in accordance to our findings that microbial communities in deepsoils are less studied compared to those of topsoils. We assume that oligotrophic microbial communities in deep soils harbor more unannotated genes than copiotrophic microbial communities. These results can be attributed to the fact that oligotrophs are less readily culturable than copiotrophs, and thus their taxonomic and functional information are underrepresented in current reference databases.

### Differentially Abundant Genes of C and N-Transforming Processes

Specific information on metabolic properties can be gained from the differentially abundant genes of the CAZy and NCyc databases. The composition of C-degradation genes can reflect the ability of microorganisms to utilize various C-complexes, thereby affecting the accumulation of SOC pools. Gene relative abundance is an adequate predictor of the associated enzyme activity (Trivedi et al., 2016).

With the CAZy database we identified a significant increase in the deep soil in genes for glycosyl transferases (GT) and the carbohydrate binding modules (CBM). The GT genes play essential roles in the biosynthesis pathways of oligo- and polysaccharides, as well as in protein glycosylation and the formation of valuable natural products, and the CBM genes are involved in the binding of carbohydrates, mainly starch, pectin, chitin, and cellulose. It has been considered that CBM genes enhance enzymatic hydrolysis, especially for insoluble substrates (Sidar et al., 2020). In addition, there was a distinct dominance of overrepresented genes in the CAZy database in the deep soil, indicating high metabolic activity of anabolic and catabolic processes. The degradation of oligosaccharides, chitin, pectin, starch, cellulose, and hemicellulose were very common and dominant in the deep soil, whereas those for degradation of lignin did not differ from the topsoil, with the exception of overrepresented genes for multicopper oxidases (AA1, e.g., laccase). It seems that lignin degradation mainly occurs in the topsoil, where most of the fungi live and are active (e.g., Žifčáková et al., 2017; Frey et al., 2021), if we consider fungi as the main lignin decomposers. Thus, we conclude that Bacteria and fungi in deep soil horizons mainly access easily degradable carbohydrates for their survival and growth, as oxygen and nutrients may be limited and at the edge of suitable concentrations (e.g., Lennon, 2020). Microorganisms cannot only promote the release of C into the atmosphere through their catabolic activities but also synthesize labile C into a stable form through their anabolic functions (Liang et al., 2017). We observed that genes for microbial anabolic activities (GTs) were more abundant in the deep soil, however, further studies are required to investigate the contribution of microbial-derived C to SOC sequestration in the deep soil.

With the NCyc database we found a significant increase in the deep soil in genes involved in organic degradation and synthesis. The significant increase in the abundance of N-fixing genes, however, can be neglected because the number of DNA reads was extremely low and, therefore, not relevant. Overrepresented genes in the deep soil were mainly involved in the degradation and synthesis of N compounds such as urea or glutamate, in nitrification and denitrification, and in nitrate reduction. Interestingly, genes involved in nitrification via ammonia monooxygenases (*amo*) were dominant, with all three subunit types (*A, B, C*) of Archaea and two subunit types (*B, C*) of Bacteria overrepresented in the deep soil. It has previously been reported that ammonia-oxidizing Archaea rather prefer areas with low ammonium and have a higher survival rate under conditions of low oxygen than ammonia-oxidizing Bacteria (He et al., 2018). This finding was also confirmed by our quantitative PCR analysis, which indicated that the deep soil harbors significantly higher gene abundances of archaeal *amoA*. However, such strong seasonal shifts in ammonia and pH are not expected in the investigated calcareous deep soil layers.

In the deep soil, genes for both assimilatory and dissimilatory nitrate reduction were overrepresented. Thus, nitrate is used not only for the assimilation but also for the conservation of energy, indicating that it is used as an electron acceptor for respiration in the (near) absence of oxygen (Kamp et al., 2015). Moreover, dissimilatory nitrate reduction and nitrate storage are physiological life traits that provide the microorganisms with flexibility and resource independence when environments are temporarily anoxic and/or nitrate free (Kamp et al., 2015). Clearly, the overrepresentation of the dissimilatory pathway is an indication of environments that are limited in oxygen even that the investigated soils did not show redoximorphic features. Consequently, microorganisms living in deeper soil horizons have to put more efforts into the mobilization, transformation, and formation of N compounds, which results in greater abundance of genes which can use nitrate not only as a N source but also in energy conservation.

Overall, there are several indications that soils in deep layers may be limited in oxygen and therefore represent a challenging environment for oxic-adapted microbial life (MacDonald et al., 1993). Among the functional genes involved in methane cycling, no methyl-coenzyme M reductase genes (the primary gene involved in methanogenesis, *mcrA*) were detected in the metagenomes. By using quantitative PCR, however, we found an overall low abundance of *mcrA* genes at both soil depths, with a higher abundance of *mcrA* genes in the topsoil. Methanogens use CO_2_ as an electron acceptor during anaerobic respiration and produce methane. Therefore, these low abundances of *mcrA* genes could indicate oxygen-limited but not fully anaerobic conditions (Frey et al., 2011). This corresponds to the findings that the abundance of the gene for methane monooxygenase (*pmoA*), an enzyme that metabolizes methane for C-metabolism and energy, was also significantly less abundant in the deep soil. Our findings suggest that methane as a substrate for *pmoA* may not be produced at higher concentrations in the deep soils studied, although methane could be oxidized anaerobically using alternative electron acceptors, e.g., nitrite, nitrate, and ferric iron (Cai et al., 2018; Shen et al., 2019).

As no measurements of oxygen concentrations in topsoils and deepsoils were carried out, one can only speculate about the interpretation of the complex interactions between methane-cycling organisms, AOA and oxygen concentrations. However, two points need to be considered. Firstly, due to the sandy and well-drained soils in deep soils, it can be assumed that deepsoils are not strongly oxygen-limited and therefore contain a lower abundance of *mcrA* genes but a higher abundance of AOA due to the more oligotrophic conditions. Such a condition is not unusual. Secondly, due to the higher microbial activity in topsoils resulting in higher oxygen consumption, and in combination with a high clay content, there are more anaerobic microniches in the topsoil. Thus, a higher abundance of *mcrA* genes can be expected in topsoils. As for methanogens, which are strictly anaerobic organisms, the presence of *mcrA* genes has previously been shown in oxic soils in anaerobic microniches (Frey et al., 2011; Li et al., 2021). In addition, anaerobic methanotrophs have been reported that can oxidize methane produced by methanogens (Luesken et al., 2012). Anaerobic methanotrophs might be present in the deeper soil layer, most likely due to their sensitivity to oxygen. This also explains the relative high abundance of *pmoA* genes in both top and deep soils.

## Conclusion

The metagenomic analysis of deep soil layers of beech and oak forests in Switzerland revealed that tree genus and forest site factors have only a minor effect on the composition of the microbial gene repertoire, while soil depth had strong effects. Soil depth decisively changes chemical conditions and resource availability leading to more favorable conditions for Archaea than for Bacteria. The strong increase in the archaeal domains in the deep soil layers also indicates that biogeochemical processes and cycling are likely to be affected by soil depth. In deep soils, genes for carbohydrate-active enzymes involved in catalyzing the transfer of saccharide moieties are overrepresented, as are enzymes involved in binding carbohydrates such as chitin or cellulose. The greater abundance of these genes in the deep soil demonstrates that greater efforts have been made for enzymatic hydrolysis, especially for insoluble substrates. Furthermore, overrepresented genes of the N cycle in the deep soils are involved in the degradation and synthesis of N compounds, in nitrification, in denitrification, and in nitrate reduction. Consequently, the entire N-cycle is affected and N is not only used for assimilation but also for energy conservation, indicating conditions of low oxygen, even if the soils were not hydromorphic. Overall, metagenomics proved to be an appropriate approach to gain insights into biogeochemical processes that may change with altered soil properties, such as those associated with greater soil depths. Our study is one of the first to offer insight into the functional diversity of heterogeneous microbial assemblages in deep forest soils. Our data on the functional genetic potential of the soil microbiomes in deep soils provide information about their metabolic capacities, enabling modeling of depth-specific biogeochemical processes that may change as a result of altered soil conditions.

## Data Availability Statement

The datasets presented in this study can be found in online repositories. The names of the repository/repositories and accession number(s) can be found below: https://www.ncbi.nlm.nih.gov/bioproject/783873, bioproject/PRJNA783873.

## Author Contributions

BF and IB designed the microbial study and wrote the main parts of the manuscript. LW established and maintained the forest sites. LW and IB provided soil and fine-root data. BS and WQ performed genetic analyses in the lab. BF, GV, and IB performed statistical analyses. All authors contributed to the final version of the manuscript.

## Conflict of Interest

The authors declare that the research was conducted in the absence of any commercial or financial relationships that could be construed as a potential conflict of interest.

## Publisher’s Note

All claims expressed in this article are solely those of the authors and do not necessarily represent those of their affiliated organizations, or those of the publisher, the editors and the reviewers. Any product that may be evaluated in this article, or claim that may be made by its manufacturer, is not guaranteed or endorsed by the publisher.

## Footnotes

1
https://www.bioinformatics.babraham.ac.uk/projects/fastqc/
