# Supplementary material for: Shotgun Metagenomics of Deep Forest Soil Layers Show Evidence of Altered Microbial Genetic Potential for Biogeochemical Cycling
Source: Front Microbiol. 2022 Mar 1;13:828977. doi: 10.3389/fmicb.2022.828977 (PMC8921678; doi:10.3389/fmicb.2022.828977)
Supplement: Supplementary file 1 [file Data_Sheet_1.PDF]

Supplementary Material

Supplementary Figures

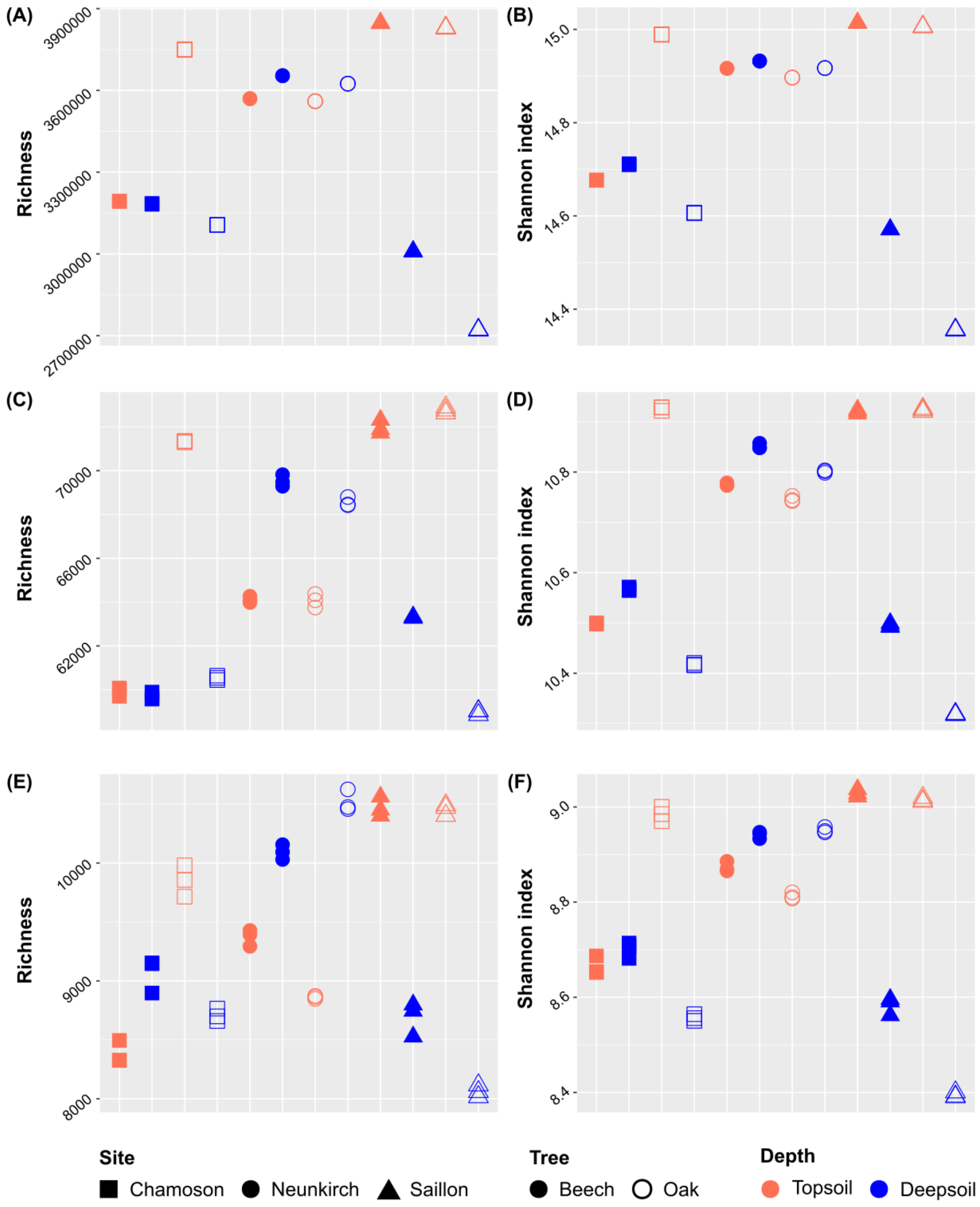

Figure S1. Alpha diversity (Richness and Shannon index) of protein-coding genes calculated over (A, B) the entire dataset, (C, D) the CAZy dataset, and (E, F) the NCyc dataset.

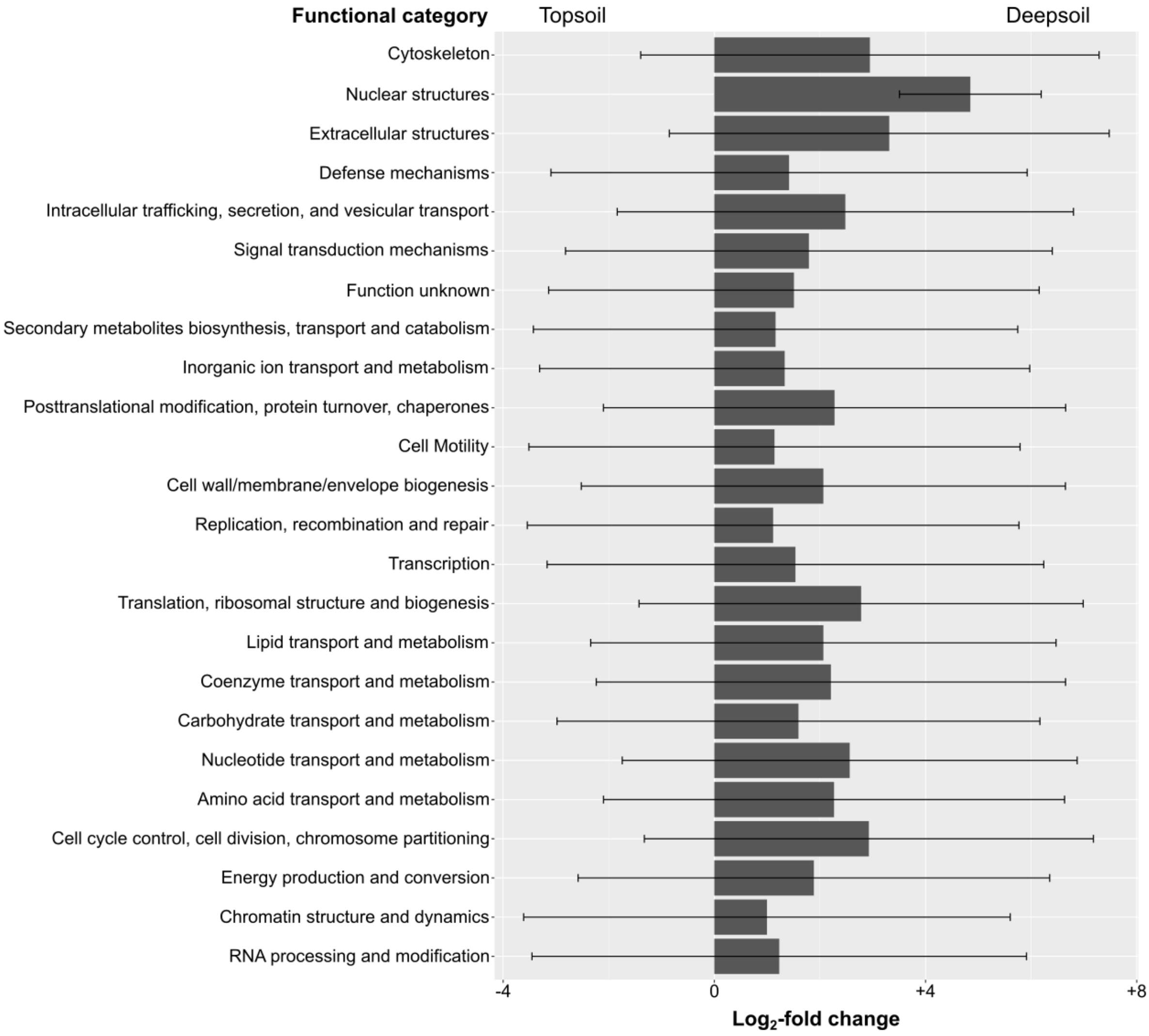

Figure S2. Over- and underrepresented genes annotated to the EggNOG database for the pairwise comparison of topsoil *vs*. deepsoil. Only significantly (P < 0.01) differentially abundant genes whose $\log_2$-fold change was lower than -1 or higher than +1 are displayed.

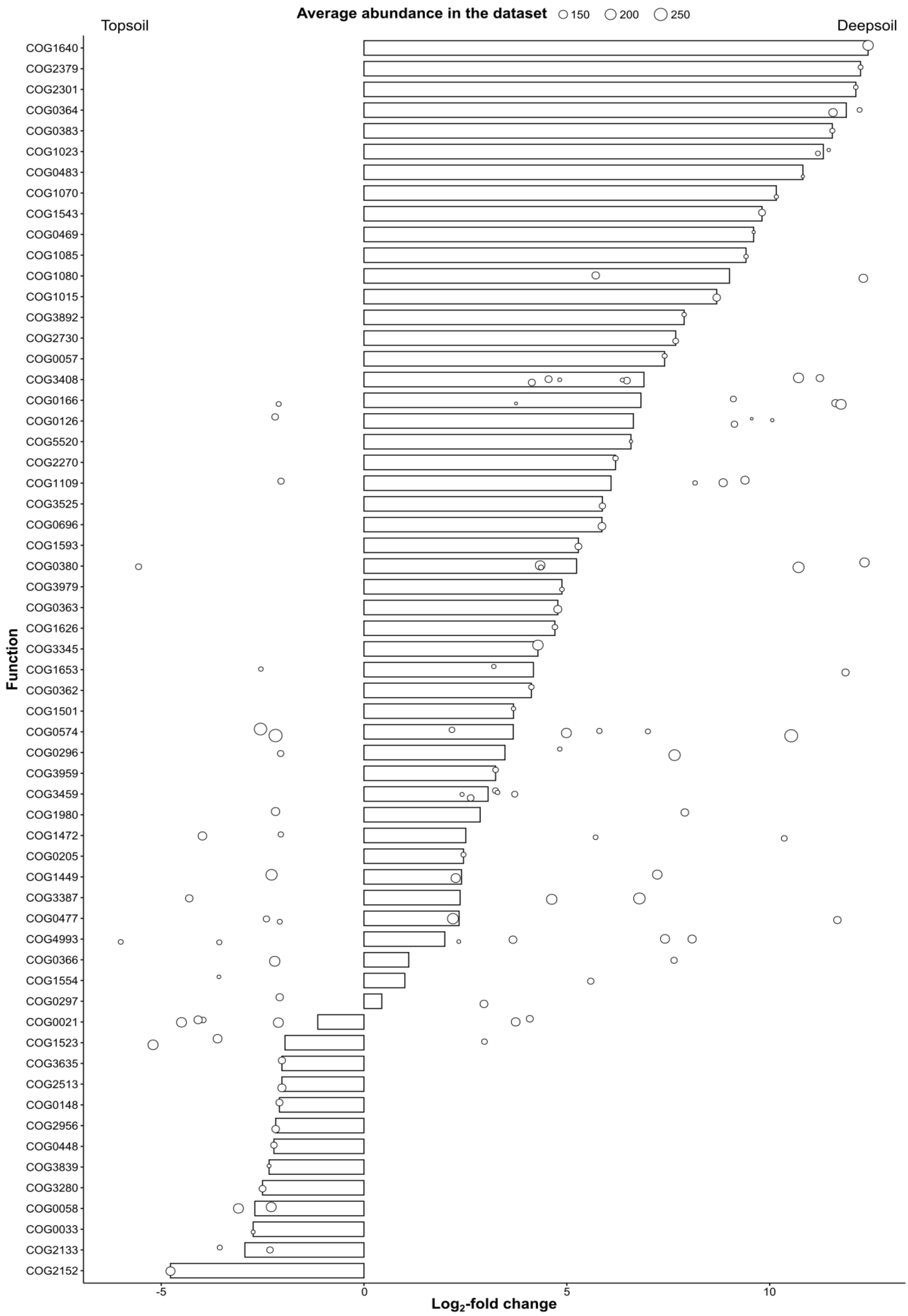

Figure S3. Over- and underrepresented functional categories of significantly (*P* < 0.01) differentially abundant genes assigned to the EggNOG database, category carbohydrate transport and metabolism (G), for the pairwise comparison topsoil *vs.* deepsoil.

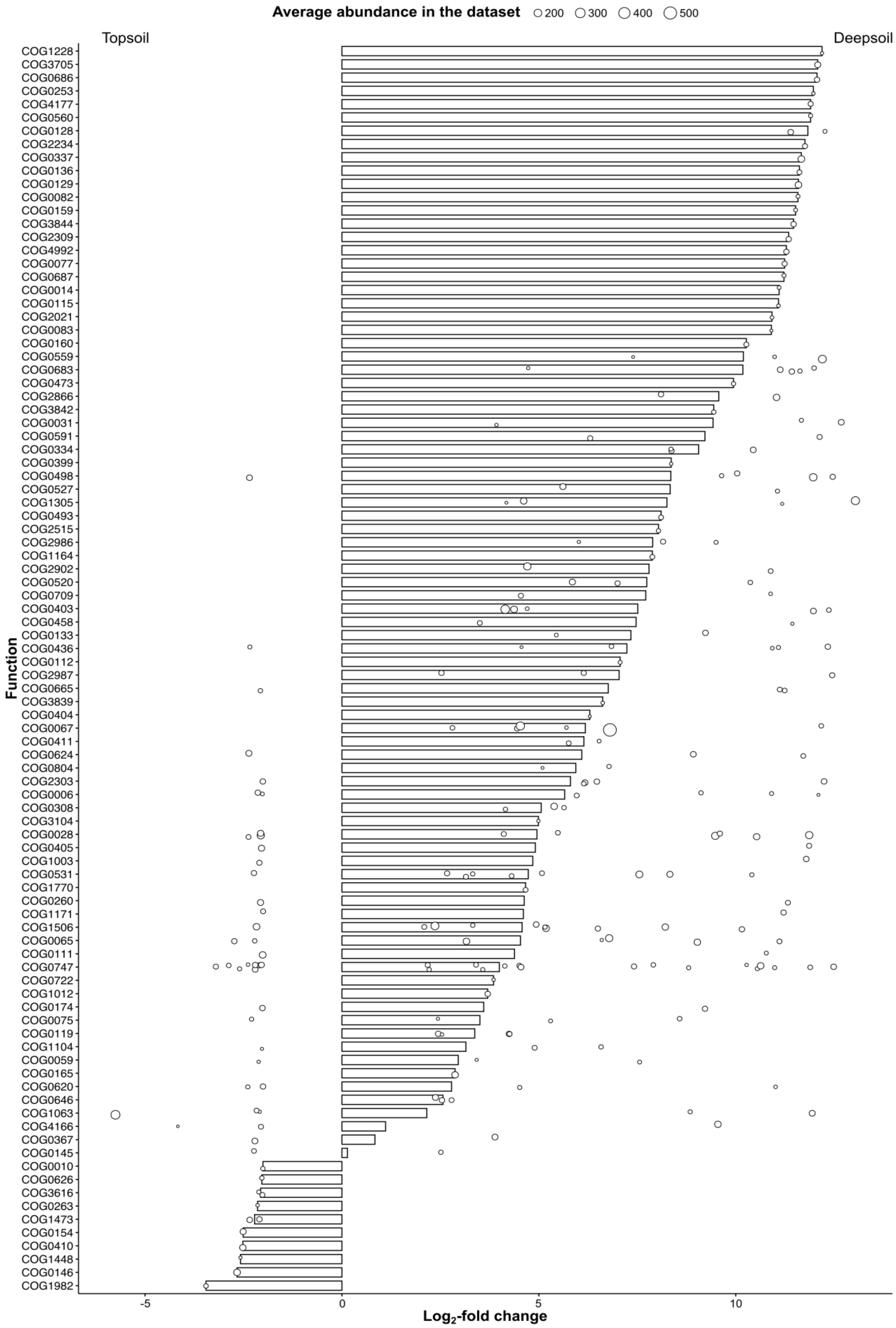

Figure S4. Over- and underrepresented functional categories of significantly ($P < 0.01$) differentially abundant genes assigned to the EggNOG database, category amino acid transport and metabolism (E), for the pairwise comparison topsoil *vs.* deepsoil.

Supplementary Tables

Table S1. Forest sites, tree and soil properties (from Frey et al., 2021)

	Lat.	Long.	Topography			Climate		Trees			Soil type
			Elev.	Exp.	Slope	MAT	MAP	Age	Height	DBH	FAO class.
	N	E	m a.s.l.		%	°C	mm	yr	m	cm	
**Beech sites:**											
Chamoson	46°12’	07°12’	880	NE	65	8.5	870	74	29	39	Eutric Cambisol
Neunkirch	47°41	08°32’	560	N	58	8.8	953	166	24	66	Rendzic Leptosol
Saillon	46°10	07°09’	890	SE	55	9.4	829	100	24	38	Calcaric Regosol
**Oak sites:**											
Chamoson	46°12’	07°12’	870	S	90	8.6	815	66	9	19	Calcaric Regosol
Neunkirch	47°40	08°32’	640	S	35	9.1	949	134	17	38	Rendzic Leptosol
Saillon	46°10	07°09’	790	SE	50	9.4	828	97	18	28	Calcaric Regosol

Lat.: latitude; Long.: longitude; Elev.: elevation; Exp.: exposition: MAT: mean annual temperature; MAP: mean annual precipitation sum; DBH: stem diameter at breast height (mean of the three sampled trees); FAO class.: classification according to IUSS Working Group WRB. 2007. World Reference Base for Soil Resources 2006, first update 2007. World Soil Resources Reports No. 103. FAO, Rome.

Table S2. Statistics of the genome assembly.

Parameter	Size
Assembly size (bp[+])	21,073,237,622
Number of high quality reads after filtering	1,642,428,601
Number of MEGAHIT contigs	37,083,277
Maximum contig length (bp[+])	409,562
Minimum contig length (bp)	202
Mean contig length (bp)	568
Median contig length (bp)	9,663
N_{50} contig length (bp)	581
GC content (%)	64
Number of predicted protein-coding genes with MetaGeneMark[†]	50,837,701
Number of predicted protein-coding genes annotated with EggNOG database[*]	22,941,682
Number of predicted protein-coding genes annotated with CAZy database[#]	507,956
Number of predicted protein-coding genes annotated with NCyc database[‡]	82,022

[+] bp: base pairs.

[†] MetaGeneMark: Tool for gene prediction in metagenomes, utilizing metagenome parameters and gene prediction.

[*] EggNOG: Database of orthology relationships, functional annotation, and gene evolutionary histories.

[#] CAZy: Carbohydrate-active enzymes database.

[‡] NCyc: Curated integrative database for fast and accurate metagenomic profiling of nitrogen cycling genes.

Table S3. Statistics of alpha diversity (Richness and Shannon index) of protein-coding genes calculated over the entire dataset of essential genes, the CAZy dataset, and the NCyc dataset.

			Richness			Shannon index		
		DF#	Pseudo-*F*	R^2	*P*	Pseudo-*F*	R^2	*P*
All genes:	Site	2	2.64	0.14	0.08	3.30	0.17	**0.048**
	Tree	1	0.04	0.00	0.85	0.01	0.00	0.91
	Depth	1	17.74	0.34	**<0.001**	17.23	0.34	**<0.001**
CAZy genes:	Site	2	2.46	0.13	0.10	2.86	0.15	0.07
	Tree	1	0.58	0.02	0.45	0.00	0.00	0.95
	Depth	1	6.33	0.16	**0.016**	13.94	0.29	**<0.001**
NCyc genes	Site	2	2.25	0.12	0.12	2.56	0.13	0.10
	Tree	1	0.05	0.00	0.82	0.03	0.00	0.86
	Depth	1	1.79	0.05	0.19	12.67	0.27	**<0.001**

Values represent degrees of freedom (DF), F-value (Pseudo-F), strength of the correlation (R^2), and the level of significance (P); significant values (P < 0.05) are in bold.

Table S4. Number of genes of the EggNOG database (orthologous proteins and functional annotations at multiple taxonomical levels).

	Beech sites		Oak sites		*P*-ANOVA+		
	Topsoil	Deepsoil	Topsoil	Deepsoil	Tree	Depth	Inter.
**Information storage and processing genes:**							
RNA processing (A) ($x10^6$)	0.06	0.06	0.06	0.06	0.61	0.77	0.76
Chromatin structure (B) ($x10^6$)	0.02	0.02	0.02	0.02	0.93	0.57	0.44
Translation, ribosomal (J) ($x10^6$)	5.92	7.75	5.56	8.29	0.87	**0.002**	0.42
Transcription (K) ($x10^6$)	7.92	8.57	7.99	9.81	0.32	0.08	0.36
Replication, recombination (L) ($x10^6$)	11.93	12.38	11.23	12.76	0.87	0.24	0.52
**Cellular processes and signaling genes:**							
Cell cycle control (D) ($x10^6$)	1.05	1.45	1.01	1.57	0.73	**0.002**	0.52
Cell wall/membrane (M) ($x10^6$)	10.36	12.74	9.56	13.39	0.94	**0.016**	0.50
Cell motility (N) ($x10^6$)	0.42	0.29	0.41	0.31	0.79	**0.016**	0.76
Posttranslational modification (O) ($x10^6$)	6.66	7.98	6.19	8.39	0.95	**0.008**	0.41
Signal transduction (T) ($x10^6$)	12.24	13.72	11.37	14.16	0.79	**0.026**	0.43
Intracellular trafficking (U) ($x10^6$)	2.34	2.71	2.12	2.82	0.67	**0.006**	0.28
Defense mechanisms (V) ($x10^6$)	5.38	5.20	5.07	5.31	0.67	0.89	0.39
Extracellular structures (W) ($x10^6$)	0.016	0.023	0.014	0.025	0.96	**0.016**	0.55
Nuclear structures (Y) ($x10^6$)	0.0001	0.0001	0.0001	0.0001	0.90	0.44	0.51
Cytoskeleton (Z) ($x10^6$)	0.06	0.08	0.05	0.09	0.97	**0.024**	0.42
**Metabolism genes:**							
Energy production (C) ($x10^6$)	14.34	15.33	13.44	16.16	0.96	**0.035**	0.41
Amino acid transport (E) ($x10^6$)	15.73	18.52	14.75	19.73	0.92	**0.007**	0.34
Nucleotide transport (F) ($x10^6$)	3.31	4.25	3.15	4.60	0.74	**0.003**	0.39
Carbohydrate transport (G) ($x10^6$)	10.58	11.05	10.23	12.05	0.63	0.11	0.33
Coenzyme transport (H) ($x10^6$)	4.46	5.50	4.26	5.95	0.74	**0.005**	0.40
Lipid transport (I) ($x10^6$)	6.16	6.93	5.93	7.58	0.63	**0.020**	0.32
Inorganic ion transport (P) ($x10^6$)	10.98	10.86	10.54	11.40	0.91	0.45	0.32
Secondary metabolites (Q) ($x10^6$)	5.56	5.49	5.26	5.90	0.87	0.41	0.32
**Poorly characterized genes:**							
Function unknown (S) ($x10^6$)	43.81	46.35	40.95	48.38	0.87	0.08	0.35

+ *Effects of tree genus, soil depth, and their interaction were assessed by analysis of variance (ANOVA); significant values (P < 0.05) are in bold.*
